# Introducing Parenting Support in Primary Care: Professionals’ Perspectives on the Implementation of a Positive Parenting Program

**DOI:** 10.1007/s10935-021-00664-x

**Published:** 2022-01-06

**Authors:** Enrique Callejas, Sonia Byrne, María José Rodrigo

**Affiliations:** Department of Developmental and Educational Psychology, Facultad de Psicología, Universidad de La Laguna, Campus de Guajara. 38071 La Laguna, S/C de Tenerife, La Laguna, Canary Islands, Spain

**Keywords:** Parenting program, Evidence-based parenting support, Primary healthcare, Pediatrics, Early childhood

## Abstract

While positive parenting programs are an initiative aligned with the Family-Centered Care model and the Council of Europe’s Recommendation on Positive Parenting, implementation in healthcare centers remains a challenge. The aims of this study were to (1) investigate how the hybrid version (online course plus face-to-face activities) of the program “Gain Health & Wellbeing From 0 to 3” was implemented in Spain from professionals’ perspective, and (2) explore the perceived impact of this hybrid version of the program on the implementers’ professional development. We used a qualitative mixed-methods design that included focus groups and surveys. Fifty professionals from 17 centers completed the survey on professional development. Thirty-one of these also participated in the focus groups to address the first aim. The key themes identified from the focus group were professional training, parent recruitment, program features, organizational issues, parental responses, and program sustainability. Survey results related to positive professional impact fit nicely with subthemes concerning collaboration with parents, parental needs, center coordination, and future expectations. The perceived relevance of the parenting program and its positive impact on the implementers’ professional development were potential predictors for the adoption and sustainability of the program in the public health system.

## Introduction

Most Primary Healthcare centers have begun to pay attention to the Family-Centered Care (FCC) model (Shields, 2015). FCC is characterized by a professional-family relationship based on mutual respect, information sharing, and joint responsibility for the child’s healthcare (Fordham, Gibson, & Bowes, 2012). When applied to early childhood, this model considers parental figures as one of the main contributors to their child’s health and wellbeing (Bellis et al., 2017). Thus, guiding and supporting parental figures as health promoters should be part of professionals’ regular practice in pediatric settings.

However, the introduction of the FCC model into primary healthcare centers remains a challenge. First, pediatrician training programs, as compared to nursing and social work programs, have paid little attention to psychosocial aspects of child health, such as care and education in the family context (Briggs, 2016). Second, some of the strategies to promote healthy practices are still far from a collaborative approach (Michie, 2008). Third, integrating parenting support entails the risk of not finding sufficient space in the professionals’ work schedule, potentially leading to situations of burnout (Rudebeck, 2019). Finally, healthcare professionals are generally not familiar with evidence-based positive parenting programs as the most appropriate way to deliver parenting support (Leslie et al., 2016). Despite the opportunity offered by the FCC model, a recent meta-analysis of primary care-based interventions (aimed at parents of children younger than 36 months) identified 13 programs with less than half aimed at promoting positive parenting behaviors (Shah et al., 2016). The parenting programs reviewed were primarily delivered through face-to-face activities at group meetings (e.g., Triple P: Sanders & Turner, 2019; Incredible Years: Webster-Stratton, 2016) and, to a lesser extent, through one-on-one consultations at pediatric check-ups and home visits (e.g., Incredible Years Well Baby Program: Pontoppidan, Klest, & Sandoy, 2016; Healthy Steps: Briggs, 2016). Half of these interventions used pediatricians, whereas the rest incorporated other healthcare professionals or even external specialists, which in the latter case may have jeopardized program sustainability. To overcome this barrier, some countries (e.g., the United Kingdom and the Netherlands) integrated comprehensive parenting support into their primary care centers through programs such as the Family Nurse Partnership (Asmussen & Brims, 2018; Mejdoubi et al., 2015), implemented by primary care providers.

The Spanish Ministry of Health sponsored the positive parenting program “Ganar Salud y Bienestar de 0 a 3 Años” (“Gain Health & Wellbeing From 0 to 3 Years,” or GH&W; Rodrigo et al., 2017), and encouraged its use by the public pediatric centers. This initiative is in line with the Council of Europe’s recommendation on policy to Support Positive Parenting (Council of Europe, 2006), which pointed out the need for the public health system, among other sectors, to provide universal parenting support. As a prevention strategy, the GH&W program has three versions: (a) an online version aimed at universal populations, (b) a hybrid version (online version plus face-to-face support) aimed at both universal and selective populations, and (c) a face-to-face version aimed at specific populations. The online version has reached a high level of satisfaction among parents and professionals (Callejas, Byrne, & Rodrigo, 2018). The hybrid version has shown good feasibility and effectiveness (Callejas, Byrne, & Rodrigo, 2021). The face-to-face version is currently under evaluation in a clinical sample of mothers with mental health problems attending a hospital perinatal unit. The GH&W program is based on the developmental system model, which focuses on the development of stimulating daily routines and healthy parent-child activities (Guralnick, 2013). This is considered a way to empower parental figures to become active agents of their child’s health. The online version of the GH&W program includes four modules designed to foster a child’s attachment, food, play, and healthy sleeping habits. Each module offers parents three different training pathways, including developmental milestones and educational strategies according to the child’s age (0–1 year old, 1–2 years old, and 2–3 years old). The program utilizes an experiential methodology (Callejas et al., 2018) that presents everyday parent-child situations (using videoclips, illustrations, and interactive materials) displaying parental attitudes and behaviors on which parents are asked to reflect. The full program in its online version takes an average of eight hours to complete, and is hosted in a Moodle platform, freely available at http://aulaparentalidad-msssi.com/.

Despite the increasing relevance of hybrid parenting programs, little is known about the conditions that assure their correct implementation in primary healthcare settings. The implementation process involves a broad range of variables related to the program itself (e.g., dosage, recruitment, didactic and material resources), as well as systemic-organizational variables that constitute the context of the program (e.g., training of professionals, technical assistance, organizational capacity, and the organizational culture of the center; Berkel, Mauricio, Schoenfelder, & Sandler, 2011). Recent reviews call for more research to capture emerging factors that contribute to effective implementation (Smith et al., 2020), especially those factors reported by professionals (Koerting et al., 2013).

Our study has two aims, the first of which is to qualitatively explore facilitators’ perspectives concerning the initial implementation of the hybrid version of the GH&W program carried out by health care professionals at the primary healthcare centers. Our second aim is to quantitatively explore professionals’ opinions about the impact of implementing the GH&W program on their professional development.

## Methods

### Procedure

Our study took place in 17 public primary healthcare centers that belong to the Canary Health Service in Spain. The Management of the Canary Health Service informed the network of primary healthcare centers of the study and created a list of potential centers. We selected the centers from all those that voluntarily joined the study, following a stratified random sampling procedure that considered the zone and the proportion of those serving children under three years of age. Participants included nurses, midwives, pediatricians, and social workers who were responsible for implementing the program.

We randomly assigned the centers to one of three levels of intervention. Participants in Level 1 centers (L1) agreed to promote only the use of the online version of the GH&W program through dissemination of leaflets at regular check-ups to parents who had children between 0 and 3 years of age. Participants in Level 2 centers (L2), in addition to dissemination, were asked to conduct four face-to-face group workshops following the online version content developed through experiential methodology with those parents interested in the online course. Workshops were conducted in primary healthcare centers. Participants in Level 3 centers (L3), in addition to performing the L1 and L2 activities, provided individual support at regular pediatric check-ups (one session at the beginning of the program) involving the Teachable Moment Technique, which consists of creating an event to prompt behavior change (Lawson & Flocke, 2010), using a protocol to explore parents’ concerns and perceived strengths.

All participants received specific training appropriate for the level of intervention. Participants in L1 were given a 30-min talk introducing the online version of the GH&W program to promote its use among their patients by providing flyers. Participants in L2 received a 5-hr training session on Positive Parenting principles, the content of the hybrid GH&W program (online plus group support), and the moderator skills necessary to manage the group workshop effectively. Participants in L3 received an additional 2-hr session (total 7 h) on the content of the individual support. All participants received technical assistance and supervision throughout the process. After the initial training, participants recruited parents at regular check-ups and implemented the program twice.

For the focus group, only participants in L2 (*n* = 12) and L3 (*n* = 19) participated, since they were the ones who had implemented the program, whereas the survey involved the full sample of participants in L1 (*n* = 12), L2 (*n* = 16), and L3 (*n* = 22).

### Participants

We initially contacted a total of 67 healthcare professionals from 20 centers. The final sample consisted of 50 participants from 17 centers who completed the survey, and of those, 31 also participated in a focus group. The participants were very similar professionally across the qualitative and quantitative phases. The ages of the participants ranged from 26 to 61 years (average of 45), of whom 45 identified as female, 25 reported having conducted parenting activities, 23 had a bachelor’s degree, 26 a master’s degree, and one a doctorate. The participants reported an average of 20 years of professional experience. There were more nurses (*n* = 26) and pediatricians (*n* = 20) than social workers (*n* = 4). Participants’ demographics did not differ significantly across the three levels of intervention described in the procedure.

All participants provided informed consent. Participants joined the study voluntarily without receiving any compensation. Participating in the study involved completing a general questionnaire, implementing the program, completing a professional development survey, and possibly taking part in a focus group.

### Instruments

#### Participant General Information

This form consisted of six items. Participants reported their sex, age, academic level (bachelor’s or graduate degrees), position, years of experience as a practitioner, and whether or not they had experience running parenting interventions.

#### Impact on Professional Development Survey

We adapted this survey from the one used for face-to-face parenting programs (Byrne, Rodrigo, & Máiquez, 2014) which included 18 items addressing the impact of the program at the individual, team, and center levels. Participants rated their agreement with each statement on a Likert scale (1 = Strongly disagree to 5 = Strongly agree) that assessed: (a) Impact on individual work with families (6 items, α = 0.89; “*I have more skills to help me understand what happens to families*”); (b) Impact on teamwork (5 items; α = 0.84; “*The experience has allowed me to get to know other professionals better*”); and, (c) Impact on center organization (7 items; α = 0.64; “*The GH&W program has been integrated into the health promotion actions of the center*”). The score of the subscales corresponded to the mean score of their items (1–5 scale). Higher scores indicated a more positive impact on individual, teamwork, and healthcare office professional development, respectively.

#### Moderator’s Guide

The purpose of the guide for the focus groups with participants was to generate information about the implementation process and the program’s impact on professional development. The moderator’s guide tackled these two topics through open-ended questions (see Table 1).

**Table 1:** Moderator’s guide

Topics	Subtopics	Questions
Introduction	Introduce the aim and the structure of the focus group session	The aim of this interview is to know your point of view about the program and its implementation. The focus group will last an hour approximately. We will discuss the process, from the beginning (training) to the end (sessions with families) and the changes that you have had throughout this experience
Implementation	Training	Which strategies have you learned thanks to the training? What contents would you add to the training sessions?
	Recruitment and engagement	What do you think motivates families to attend the sessions? What are the barriers that hinder families’ attendance?
	Sessions	What do you value most in the sessions? Which activities have you felt more comfortable working with? How confident have you felt implementing the group sessions?
	Implementation requirements	Which conditions would be necessary to implement the program as a regular practice at your center (resources, facilities, etc.)?
	Improvement suggestions	If the sessions restarted, what would you do differently? What would you keep doing?
Impact on the professional development	Learnings from this experience	Do you feel more able to identify the parents’ needs? Do you feel more able to promote healthy changes in the family routine? Has your relationship with the families changed?
	Expectations	Are you willing to repeat sessions in the future or develop health promotion activities with parents?
Close	Summary and acknowledgment	Would you want to add something else? Thank you very much

### Data Collection

We carried out the study between March and July of 2018. In March, participants completed an initial questionnaire of their general information. Between March and June, participants implemented the program with the families. In July, participants completed a final survey (Impact on professional development survey) and then the focus groups were held. The survey was completed online. Each generally took around 10 min to complete. We conducted six separate focus groups, and participants selected the one that suited them best. The focus groups had a mean size of 6 people and lasted a mean of 48 min. At the end of each focus group, the coordinator summarized the main ideas and asked participants whether this summary reflected what they had intended to express. We tape-recorded and transcribed all the focus groups.

### Data Analyses

To explore participants’ opinions on the impact that the experience had for their professional development, we analyzed their responses to the survey. For these analyses, we calculated the mean scores by accumulating the items of each aspect, since the reliabilities were adequate. Using the IBM SPSS v25, we performed ANOVAs on the mean scores corresponding to each of the three aspects surveyed (individual, teamwork, and center organization impact). We used the assignation of the participants to L1, L2, and L3 conditions as the independent variable to examine under which level of intervention participants were more likely to see a positive impact of the program on each aspect surveyed.

We used a joint display (Guetterman, Fetters, & Creswell, 2015) to integrate the quantitative results and qualitative findings, where the qualitative subthemes were arrayed with the three survey scales tapping individual, teamwork, and center impact of the program implementation.

We conducted the qualitative analysis of the focus group transcriptions using the software ATLAS.ti v8. The thematic analysis (Braun & Clarke, 2006) guided the coding of categories and analysis. We performed three categorization cycles following the data saturation criterion (Saunders et al., 2018), setting at five responses per theme and subtheme. The focus group coordinator read the transcripts to develop an initial category chart represent the key concepts of interest. A second collaborator read a sample of the transcripts and reviewed the initial chart. We then performed a second categorization cycle to identify emerging categories that reflected participants’ opinions of the implementation process regarding strengths and concerns and to explore any proposed possible solutions. Finally, we carried out a third cycle of categorization to revise the preliminary coding schema to eliminate low-frequency codes, split codes, and merged codes. A second collaborator reviewed the categories after each cycle.

## Results

### Surveys

#### Quantitative and Qualitative Findings on Professional Development

The survey scores on program impact on professional development were moderately high across the three levels of intervention (see Table 2). However, participants who delivered face-to-face parenting support (L2 and L3) perceived a greater positive impact on their teamwork and the organization of the healthcare office than those professionals who only disseminated information about the online version of GH&W (L1). No significant differences were found regarding the individual impact on the work with families.

**Table 2:** Perceived impact on professional development across the levels of intervention (1 = Strongly disagree; 5 = Strongly agree)

Variables	L1 *M* (*SD*)	L2 *M* (*SD*)	L3 *M* (*SD*)	*F* (2, 48)	Post hoc	Partial Eta Squared
Individual impact	3.28 (0.67)	3.74 (0.96)	3.69 (0.76)	1.28	1–2 1–3 2–3	0.05
Teamwork impact	3.33 (0.47)	3.77 (0.25)	3.66 (0.38)	5.65*	1–2** 1–3* 2–3	0.19
Center organization impact	3.30 (0.83)	4.14 (0.76)	4.15 (0.81)	5.97**	1–2* 1–3** 2–3	0.20

*p < .05; **p < .01

We present the results of the merged analysis using a joint display (Guetterman et al., 2015). These findings expand and corroborate the information obtained from the survey data with the focus group subthemes’ content (see Table 3). When assessing the individual impact, participants were aware of improvements in their individual collaboration with parents, in learning new topics, and in being able to detect parental needs. When assessing the impact on teamwork, participants appraised this experience as an opportunity to work together, which is not usually the case in primary care settings, where patient care predominates, and collaborative spaces are less customary due to time constraints. When assessing the impact on center organization, participants related this impact to novel coordination with other services such as social welfare, especially regarding the recruitment of parents with risk factors. Moreover, they reported positive views about the program’s sustainability at their centers and the feasibility of introducing the program into primary care settings.

**Table 3:** *Frequency and quotations of subthemes related to survey scales*

Quantitative phase	Qualitative phase		
*Survey scales*	*Emerged related categories (subthemes)*	*Illustrative quotations*	*Related quote frequency*
Individual impact	Collaboration with parents	*“It [this experience] has helped us to get closer to the families, to create a bond.”*	17
	Positive parenting and child development	*“I have learned many things, from the [online] course especially and from the extra links […] and I learned what positive parenting was, which I did not know.”*	10
	Parental needs	*“What has impacted me the most has been to see first-hand the need that parents have for information and the hunger they have to be informed.”*	6
Teamwork impact	Professional coordination	*“We don’t have the opportunity in primary care because of our workload, our team doesn’t have a reason to say, “let’s work together,” so it was also a positive experience for me to work with colleagues.”*	15
Center organization impact	Coordination with other services	*“We contacted the village kindergarten and that helped us with recruitment.”*	10
	Program feasibility in primary care settings	*“I mainly like it [GH&W] because it gets me out of what I usually do, which is basically treatment. I think we also have to do prevention.”*	24
	Future expectations about the program	*“The regular “Healthy Child Program” for all services is being updated, it [GH&W] could be included.”*	14

### Focus Groups

#### Qualitative Findings on Professionals’ Perceptions of the Implementation Process

We obtained a total of eight themes, 34 categories (subthemes) and 902 coded comments related to the implementation process and its impact on professional development (see Table 4). The most commented theme related to organizational issues, with the highest overall percentage of quotes (31.6%). The remaining themes obtained between 7% and 15% of the coded quotes, with the impact on professional development being the least quoted (3.7%). Participants requested more detailed initial information and more complete training. Participants perceived a positive response from parents, although they reported parent recruitment as one of the main challenges. In addition, they made suggestions about organizational issues and program sustainability. Finally, participants perceived that having implemented the program had helped them to improve their relationship with the parents and to bring their practice closer to the FCC model. Quotations from professionals are available upon request. We describe each theme and related subthemes in the following paragraphs.

**Table 4:** *Frequency of quotes in the categories structured by themes*

Themes	Emerged categories (subthemes)	Quotes ^a^	
I. Initial information	Content of the initial information	39	7.4%
	Source of the initial information	28	
II. Professional training	Training content	26	10.6%
	Training duration	22	
	Training delivery format	16	
	Other aspects of the training	13	
III. Parent recruitment and adherence	Parent recruitment, adherence strategies used	58	8.5%
	Suggested recruitment, and adherence strategies	38	
IV. Program features	Online version content	9	10.8%
	Online version design	11	
	Workshop session design	46	
	Adaptations made at workshop session	16	
	Program evaluation	15	
V. Organizational issues	Coordination with other services†	10	31.6%
	Professional coordination†	15	
	Experience timing	34	
	Timing of the workshop sessions	66	
	Facilitating factors	26	
	Organizational barrier factors	38	
	Professional barrier factors	10	
	Parent barrier factors	31	
	Implementation requirements†	55	
VI. Parent response	Parent expectations about the program	20	11.9%
	Parent attendance	32	
	Parent engagement	18	
	Parent satisfaction with the workshop sessions	30	
	Program effects on parents	7	
VII. Program sustainability	Program feasibility for primary care settings†	24	15.5%
	Relevance of the program	55	
	Future expectations about the program†	14	
	Professional satisfaction with the program	47	
VIII. Program impact on professional development	Collaboration with families†	17	3.7%
	Positive parenting and child development†	10	
	Parental needs†	6	

^a^Theme percentage out of a total of 902 quotations

†Marked subthemes are part of the subsequent merged analysis

##### Theme I: Initial Information

Several participants shared their perceptions of how the initial information about the parenting program was provided, including the content and source of the information. Although some were satisfied with this aspect, participants generally demanded clear initial information from the research team instead of from secondary sources (e.g., the head of the center).

##### Theme II: Professional Training

Participants shared their opinions about the content, duration, and delivery format of the training received from the research team. Participants were generally satisfied with the face-to-face format and content. Others asked for longer and more in-depth training on group dynamics.

##### Theme III: Parent Recruitment and Adherence

Some participants’ comments revealed that parent recruitment and adherence were seen as the main challenges, since the enrolment rates were lower than expected, there was low commitment to regularly attend the sessions and at-risk parents were very difficult to reach. The main recruitment context was the medical check-ups, although some professionals mentioned the use of mailing lists or phone calls. Participants linked the recruitment and adherence challenges with possible solutions such as coordination with other services (e.g., social services) or using mass media to disseminate the information.

##### Theme IV: Program Features

Most participants showed an overall satisfaction with the GH&W program design and content as well as with the workshop design. They also praised the experiential methodology based on the interactive approach. They mentioned some points for improvement, such as clarifying the interpretation of some videoclips in the GH&W program or proposing the incorporation of new activities at workshops. With regard to the program evaluation, they were not comfortable with the number of evaluation instruments given to parents.

##### Theme V: Organizational Issues

This was the theme most mentioned. They focused on professional, parent, and organizational barriers and possible solutions as well as facilitating factors for future implementation. Participants suggested the need for longer preparation periods, since time restrictions were generally seen as an organizational barrier.

Participants also identified some facilitating factors, such as the head of the center’s positive attitude towards the innovation. Participants also talked about what would be required to continue implementing the sessions. One of the most frequently mentioned points was the importance of considering the program implementation as part of their regular practice. Furthermore, they made suggestions about the convenience of holding two program editions per year involving four workshop sessions each instead of only one edition. In sum, participants pointed out lack of time, but they also made specific proposals to overcome these issues in further program implementations.

##### Theme VI: Parent Response

This theme covered mainly the participants’ reflections on the positive parent engagement in the activities, such as satisfaction with the program and high levels of engagement at the workshop sessions, their changing views about how to manage the group discussions in the workshops, and some improvements in the parents’ autonomy, who became less dependent on professionals to cope with day-to-day parenting challenges. Participants also mentioned one attendance issue related to the need to bring together a minimum number of participants to run a group following the experiential methodology, since it is based on parent participation. They stated that parents were pleasantly surprised to receive this kind of parenting support in primary care settings. Participants highlighted this aspect as one of the reasons behind the low attendance rate in certain areas.

##### Theme VII: Sustainability of the Program

Most participants commented on their concerns for the sustainability of the experience. The majority mentioned the importance of integrating parenting support into the healthcare office using programs such as GH&W. Moreover, they considered this innovation feasible if some basic requirements were to be assured, such as including parenting support activities in their work schedule. Regarding satisfaction with the experience, participants especially valued having time to devote to health prevention and not only to the treatment of problems. They were also satisfied with having participated in an innovative project, which allowed them to develop new practices, such as facilitating group workshop dynamics.

##### Theme VIII: Impact on Professional Development

Like program sustainability, participants reported that GH&W program implementation positively influenced their professional development. They pointed out improvements in their collaborative relationships with parents and in the learning of new issues regarding the positive parenting approach and psychological developmental landmarks. They also perceived themselves as more aware of parents’ needs as promoters of their children’s health.

## Discussion

In this study, we provided a comprehensive picture from the professional’s perspective of the implementation process in primary care settings of the supportive activities that corresponded to the hybrid version of the GH&W program. As for the first aim, the categories widely covered those dimensions mentioned in the Berkel and colleagues (2011) implementation model, drawn from other studies in social domains (Aarons, Sklar, Mustanski, Benbow, & Brown, 2017). We obtained categories related to the initial professional training, the way parents were recruited and their adherence, the quality of didactic and material resources, and participants’ satisfaction with the program. In addition, we identified categories regarding organizational variables that include facilitating factors and barriers. The detailed and nuanced accounts of program implementation that participants provided indicated just how complex the implementation of parenting support in healthcare settings is, and the degree to which implementation may affect program results.

Professionals praised the quality of the online version of the GH&W program, which is a factor that may also facilitate its adoption (Gagnon, Ngangue, Payne-Gagnon, & Desmartis, 2016). They reported that the GH&W program provides an efficient way to provide child development content that is useful for parents. They also valued the face-to-face activities and the experiential methodology, which helps to facilitate collaborative and supportive relationships with the parents.

Professionals also expressed some areas of concern. They mentioned parental, professional, and organizational barriers considered in previous implementation studies (Shapiro, Prinz, & Sanders, 2012). Professionals were worried about low recruitment rates in some centers, as is typical of universal prevention programs (Fleming et al., 2015; Heinrichs, Bertram, Kuschel, & Hahlweg, 2005). Interestingly, recruitment went well in those centers where professionals had previous experience in organizing activities with parents. Professionals also mentioned difficulties reaching parents with risk factors, and suggested coordinating with other services and having longer periods of recruitment. Finally, their lack of experience in managing group sessions was mentioned as a personal barrier.

Organizational issues were by far the category most frequently mentioned. The main barrier pointed out was the workload, which should be reduced by making program implementation part of the service delivery (Gray, Totsika, & Lindsay, 2018). Moreover, in line with previous studies (Levickis, McKean, Walls, & Law, 2019), professionals mentioned that a broader and more practical professional training and more time for preparation is necessary to overcome the difficulties encountered in setting up the experience.

With regard to the second aim, to learn professionals’ opinions concerning the impact of the program implementation on their professional development, findings confirmed the positive impact of the experience. In the survey, we found a moderate to high impact (from 3 to 4 on a 1–5 scale) at the individual level regardless of the level of intervention to which professionals were assigned, since all had been exposed to the introduction of the online version of the GH&W program. Moreover, in the focus groups, professionals pointed out changes in their beliefs and practices related to the key factors of parenting support in FCC: the collaborative approach, knowledge of child development and positive parenting, and the ability to identify parental needs. These findings can be explained by the experiential methodology of the program which facilitates learning about handling group dynamics to understand parental needs in addition to the children’s health issues (O’Malley et al., 2019). This is especially relevant since there is a lack of consensus on what the professional training needs should be in order to successfully engage in the FCC model (Fix et al., 2018).

Our survey results also showed that the impact of the experience at the teamwork and center levels was greater when professionals had an actual ‘hands-on’ experience with parents. These results merge with the relevance given by professionals in the focus group to organizational issues, such as integrating the program into their professional work schedule (Tran & Voyer, 2015) and the need for more effective coordination, among other issues.

Regarding limitations, we did not include the perspectives of either the Canary Health Service stakeholders or the parents, although professionals did report on how parents responded to the program. Not all L2 and L3 professionals who responded to the survey participated in the focus group session due to work restrictions. Finally, as the objectives of the study are exploratory, large-scale implementation studies are needed that also consider objective variables (e.g., cost-effectiveness) on the implementation process.

## Conclusions

We identified three predictors of success for the adoption of the hybrid version of the GH&W program: (a) professionals perceived the program as a key tool to promote child health and development, (b) they found it feasible and complementary with the topics they already cover at medical check-ups and they perceived that families were also satisfied with the program, and (c) the implementation of the program had a positive impact on their professional development. In sum, this experience can be considered a best practice to place universal parenting support at the forefront of European family policy to be applied across healthcare, educational, and social services.
